# Inactivating the lipid kinase activity of PI3KC2**β** is sufficient to rescue myotubular myopathy in mice

**DOI:** 10.1172/jci.insight.151933

**Published:** 2023-05-08

**Authors:** Xènia Massana-Muñoz, Marie Goret, Vasugi Nattarayan, David Reiss, Christine Kretz, Gaetan Chicanne, Bernard Payrastre, Bart Vanhaesebroeck, Jocelyn Laporte

**Affiliations:** 1 Institute of Genetics and Molecular and Cellular Biology (IGBMC), INSERM U1258, CNRS UMR7104, University of Strasbourg, Illkirch, France.; 2 INSERM UMR 1048, Institute of Metabolic and Cardiovascular Diseases (I2MC), Université Toulouse III Paul Sabatier, BP84225, Toulouse Cedex 04, France.; 3 UCL Cancer Institute, University College London, London, United Kingdom.

**Keywords:** Muscle Biology, Inositol phosphates, Protein kinases, Skeletal muscle

## Abstract

Phosphoinositides (PIs) are membrane lipids that regulate signal transduction and vesicular trafficking. X-linked centronuclear myopathy (XLCNM), also called myotubular myopathy, results from loss-of-function mutations in the *MTM1* gene, which encodes the myotubularin phosphatidylinositol 3-phosphate (PtdIns3P) lipid phosphatase. No therapy for this disease is currently available. Previous studies showed that loss of expression of the class II phosphoinositide 3-kinase (PI3K) PI3KC2β (PI3KC2B) protein improved the phenotypes of an XLCNM mouse model. PI3Ks are well known to have extensive scaffolding functions and the importance of the catalytic activity of this PI3K for rescue remains unclear. Here, using PI3KC2β kinase–dead mice, we show that the selective inactivation of PI3KC2β kinase activity is sufficient to fully prevent muscle atrophy and weakness, histopathology, and sarcomere and triad disorganization in *Mtm1*-knockout mice. This rescue correlates with normalization of PtdIns3P level and mTORC1 activity, a key regulator of protein synthesis and autophagy. Conversely, lack of PI3KC2β kinase activity did not rescue the histopathology of the *BIN1* autosomal CNM mouse model. Overall, these findings support the development of specific PI3KC2β kinase inhibitors to cure myotubular myopathy.

## Introduction

Centronuclear myopathies (CNMs) are rare genetic diseases associated with strong muscle weakness, with no treatment available to date (1). CNMs are due to mutations in several genes, including *BIN1* and *DNM2*, which encode the membrane remodeling proteins amphiphysin 2 and dynamin 2, respectively (2, 3); *MTM1*, which encodes the myotubularin lipid phosphatase (4); *SPEG*, which encodes striated muscle–enriched protein kinase (5); and *RYR1*, which encodes ryanodine receptor 1 that controls calcium release at the triads (6). The most severe form linked to *MTM1* mutations is X-linked CNM, also called myotubular myopathy. Patients display severe hypotonia and ventilator distress from birth onwards, leading to a drastically reduced lifespan, with skeletal muscles displaying small rounded fibers and organelle mispositioning (7). The *Mtm1*-knockout (*Mtm1*-KO) mouse faithfully reproduces the motor and histopathology defects of this disease (8).

Myotubularin dephosphorylates phosphatidylinositol 3-phosphate (PtdIns3P) and PtdIns(3,5)P_2_, generating PtdIns and PtdIns5P, respectively (9–11). Phosphoinositides (PIs) are short-lived nonabundant phospholipids implicated in signal transduction and membrane trafficking (12). PI3-kinases (PI3Ks) define a family of proteins producing 3-phosphorylated PI (13, 14). Class I PI3Ks mainly produce PtdIns(3,4,5)P_3_ and play key roles in cell growth and differentiation and in cancer. The only class III PI3K in mammals, called VPS34, produces mainly PtdIns3P and regulates endocytosis and autophagy. Class II PI3Ks convert PtdIns to PtdIns3P and PtdIns4P to PtdIns(3,4)P_2_ and are activated by a wide range of agonists, such as growth factors, G protein–coupled receptors, and adhesion molecules (13, 15–17). Among the 3 class II PI3K isoforms, PI3KC2β is ubiquitously expressed and a key controller of endosomal trafficking and mTORC1 signaling (18–20). In accordance with these roles, PI3KC2β binds to clathrin and raptor, indicating that part of its cellular functions may be independent of its PI kinase activity.

As myotubular myopathy is linked to loss of the MTM1 protein and/or activity and an increase in the levels of PtdIns3P (21–25), previous studies tested whether modulation of different PI3K isoforms could normalize PI levels in MTM1 loss-of-function models. Kiger and colleagues first showed in *Drosophila* that the class II PI3K and MTM1 orthologs coregulate PtdIns3P pools in nonmuscle cells, and that the depletion of class II, but not class III, PI3K rescued myotubularin-dependent muscle phenotypes (26, 27). Similarly, Sabha and colleagues showed in the *Mtm1*-KO mouse that removal of PI3KC2β expression in skeletal muscle or in all tissues significantly prevents and reverts its motor and histopathological defects (22). This rescue of the *Mtm1*-KO mouse was achieved by removing the full PI3KC2β protein, with heterozygous *Pik3c2b* deletion being less efficient in rescuing the *Mtm1*-KO phenotype. These studies thus identified PI3KC2β as a second modifier to the *MTM1*-related CNM after *DNM2* (28).The PI3K inhibitor wortmannin was found to partially rescue *Mtm1*-KO mouse survival and to improve the histopathology, but this compound is not suitable for human use and is a pan-PI3K inhibitor (22, 29).

At present, it remains unclear whether the lipid kinase activity of PI3KC2β is necessary or sufficient to rescue myotubular myopathy. This is important information for developing PI3KC2β kinase inhibitors for such a disease. Moreover, the effect of loss of PI3KC2β expression has not been tested in other forms of CNM, unrelated to MTM1 mutations. Here, we used a kinase-dead *Pik3c2b*–knockin mouse to mimic the effect of a specific kinase inhibitor and performed genetic crosses with *Mtm1* and *Bin1* mouse models. We found that specific inactivation of the kinase activity significantly rescued MTM1, but not BIN1, CNM phenotypes.

## Results

### Specific inhibition of PI3KC2β kinase activity rescues survival and force of Mtm1-KO mice.

**Figure 1:**
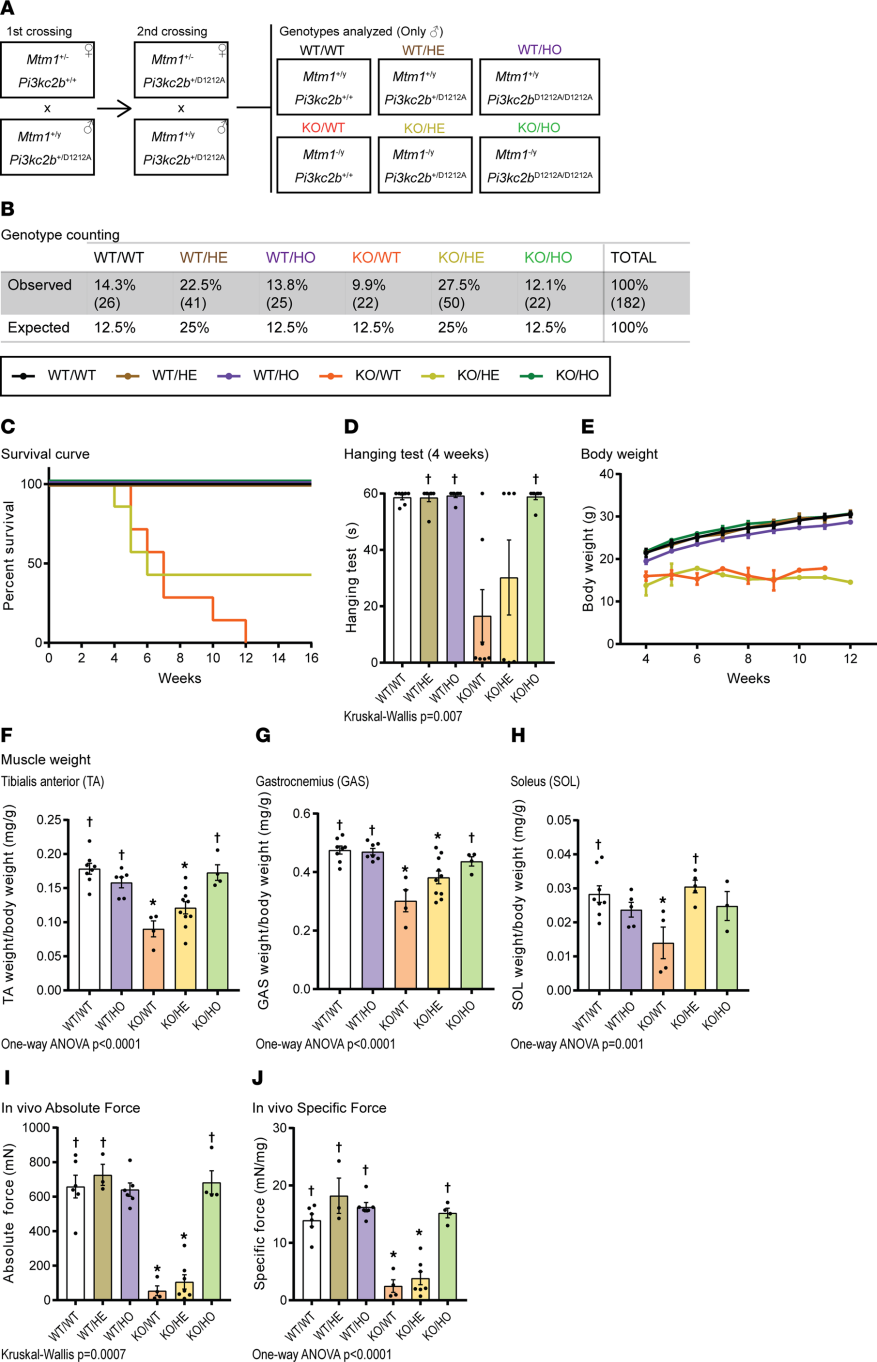
PI3KC2β kinase inhibition rescues *Mtm1^–/Y^* survival and muscle force defects. (**A**) Genotypes analyzed and nomenclature used in this study. (**B**) Percentage of genotypes obtained on postnatal day 10. (**C**) Kaplan-Meier survival curve from all genotypes analyzed (*n* = 7). (**D**) Hanging test performance at 4 weeks of age (6 ≤ *n* ≤ 7). Maximum hanging time is 60 seconds. (**E**) Growth curve representing the average body weight of each group from 4 to 12 weeks (6 ≤ *n* ≤ 7). (**F**–**H**) Muscle weight of (**F**) tibialis anterior (4 ≤ *n* ≤ 10), (**G**) gastrocnemius (4 ≤ *n* ≤ 10), and (**H**) soleus muscle (4 ≤ *n* ≤ 10) at 5 weeks. (**I**) Absolute muscle force (3 ≤ *n* ≤ 7) and (**J**) specific muscle force at 5 weeks (3 ≤ *n* ≤ 7). *P < 0.05 vs. WT/WT; ^†^*P* < 0.05 vs. KO/WT by Kruskal-Wallis test (**D** and **I**) or 1-way ANOVA test (**F**, **G**, **H**, and **J**).

To specifically inhibit PI3KC2β lipid kinase activity without affecting overall PI3KC2β protein expression, we took advantage of the *Pik3c2b*^D1212A^-knockin mouse mutated in the DFG motif of the ATP-binding domain, which inactivate the kinase activity toward phospholipids (30). In this model, only the kinase activity is abolished, while the protein is still expressed (Supplemental Figure 1A; supplemental material available online with this article; https://doi.org/10.1172/jci.insight.151933DS1). Homozygous *Pik3c2b*^D1212A^ kinase-dead mice are viable and healthy and display enhanced insulin sensitivity and glucose tolerance, as well as protection against high-fat-diet–induced liver steatosis (30). We generated 6 groups of mice: 3 control groups wild-type for *Mtm1* combined with wild-type (WT/WT), heterozygous (WT/HE) or homozygous (WT/HO) *Pik3c2b*^D1212A^, *Mtm1^–/Y^* KO mice as a disease group (KO/WT), and *Mtm1^–/Y^* either heterozygous (KO/HE) or homozygous (KO/HO) for *Pik3c2b*^D1212A^ (Figure 1A). DNA genotyping revealed the expected normal Mendelian ratio for both KO/HE and KO/HO mice, which was confirmed by Sanger sequencing (Figure 1B and Supplemental Figure 1B). KO/WT mice developed a progressive myopathy from 3 weeks of age, leading to death by 12 weeks with a median survival of 7 weeks (Figure 1C). Strikingly, all KO/HO homozygous kinase-dead mice survived up to the end of the study at 16 weeks. In comparison, 43% KO/HE heterozygous mice survived to 16 weeks. The general behavior of KO/HO mice was indistinguishable from controls and unlike the decreased activity of KO/WT mice (Supplemental Video 1). Their motor function was tested by assessing their hanging ability at 4 weeks; while KO/WT mice performed poorly (hanging time of 16 seconds), the KO/HE mice achieved 30 seconds and the KO/HO mice reached the 60-second maximum tested time similar to all control groups (Figure 1D and Supplemental Video 2). KO/HO mice displayed similar body weight as all controls, from 28 to 30 g, while KO/WT and KO/HE mice did not weigh more than 15 g (Figure 1E). The weight of different muscles, such as the tibialis anterior (TA), gastrocnemius (GAS), and soleus (SOL) was decreased in KO/WT mice and fully rescued in KO/HO mice (Figure 1, F–H). Muscle weight in the KO/HE mice was not rescued in the TA and GAS, supporting the notion that the strong decrease in body weight in the KO/WT and KO/HE mice is mainly due to general muscle atrophy. The absolute and specific forces of isolated TA muscles were strongly decreased in KO/WT and KO/HE mice and rescued to control levels in KO/HO mice (Figure 1, I and J). Overall, the homozygous *Pik3c2b* kinase-dead mutation extents the lifespan and normalizes muscle atrophy and muscle force of the myotubular myopathy KO/WT mice.

### Expression of kinase-dead PI3KC2β recovers the CNM histopathological hallmarks of Mtm1-KO mice.

**Figure 2:**
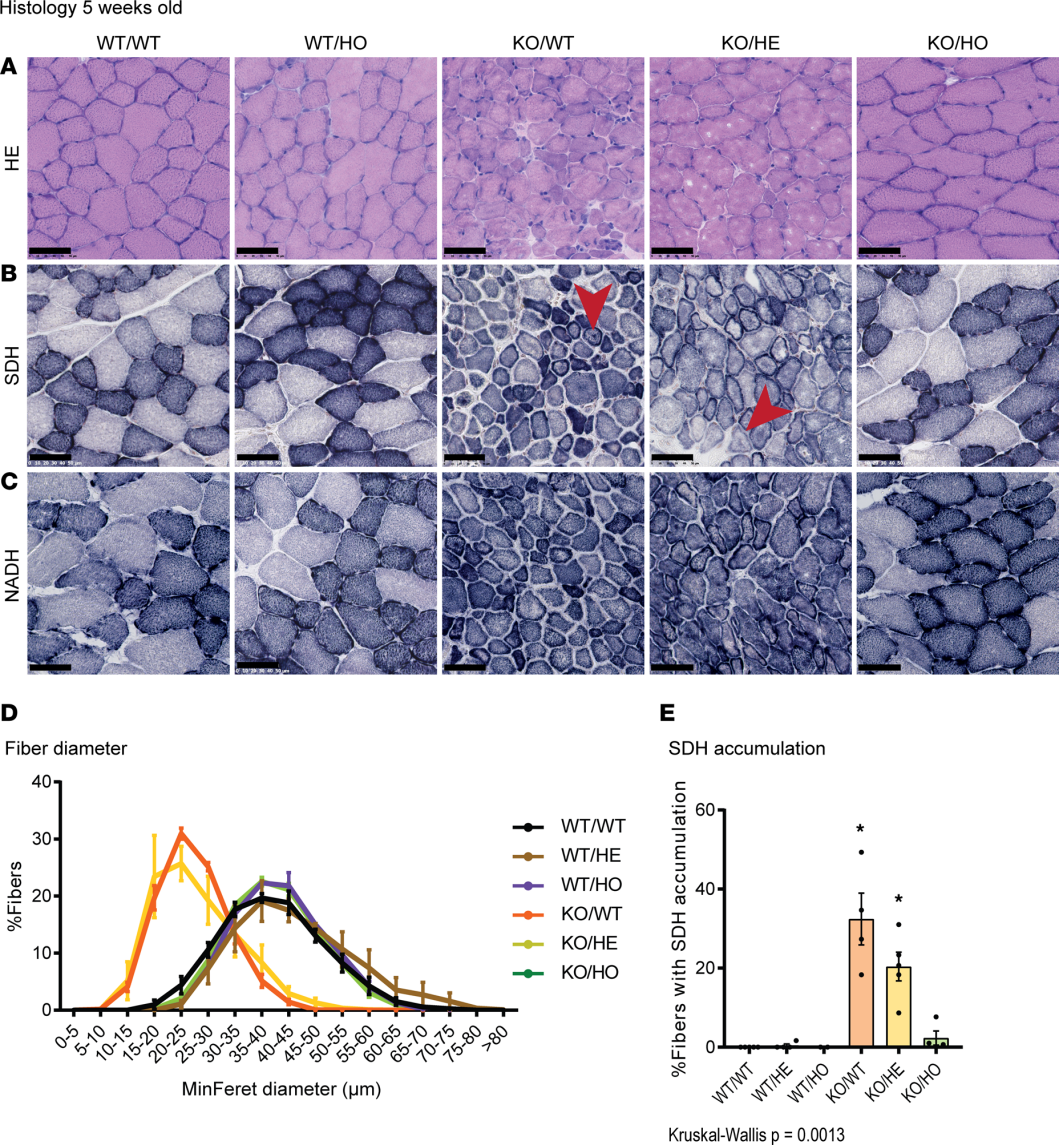
Inhibiting PI3KC2β activity rescues *Mtm1^–/Y^* muscle histological defects. Muscle sections stained with (**A**) H&E or for (**B**) SDH or (**C**) NADH. Scale bars: 50 μm. (**D**) Quantification of fiber diameter (2 ≤ *n* ≤ 4). (**E**) Percentage of fibers with central accumulation of SDH staining (2 ≤ *n* ≤ 5). All mice analyzed at 5 weeks of age. Note the central accumulation indicated by red arrowheads in **B**. *P < 0.05 vs. WT/WT by Kruskal-Wallis test (**E**).

As specific inhibition of PI3KC2β kinase activity fully rescues the muscle atrophy and force defects of *Mtm1*-KO mice, we investigated these aspects of myofibers. At 5 weeks, the *Mtm1*-KO mice display reduced fiber size with abnormal accumulation of oxidative staining, reminiscent of the histopathological hallmarks observed in myotubular myopathy patients (Figure 2, A–C). Hematoxylin and eosin (H&E) staining revealed a normal myofiber pattern and Feret diameter in the KO/HO mice (Figure 2, A and D). Similarly, oxidative activity defects were prevented in the KO/HO mice, as shown by succinate dehydrogenase (SDH) and nicotinamide adenine dinucleotide dehydrogenase–tetrazolium reductase (NADH-TR) staining (Figure 2, B and C). Specifically, the percentage of fibers with central accumulation of SDH staining was normalized from 32.4% to 2.3% in KO/HO mice compared with 0% in controls, reflecting the correction of mitochondria mispositioning (Figure 2E). At this age, no significant improvement was seen at the histological level in the KO/HE mice. Thus, specific inhibition of PI3KC2β activity fully rescues the histological phenotypes of *Mtm1*-KO mice at this age. At 16 weeks, when all KO/WT mice had died, KO/HO mice presented with slight histological defects with a decrease in the number of very large myofibers and a few fibers with central accumulation of SDH oxidative staining (Supplemental Figure 2). Otherwise, the number of small fibers and the NADH-TR staining were normal in KO/HO mice. The surviving KO/HE mice were similarly affected at 5 weeks.

In conclusion, the homozygous *Pik3c2b* kinase-dead mutation strongly delays the appearance of the CNM histology of *Mtm1*-KO mice.

### Expression of kinase-dead PI3KC2β normalizes sarcomere and triad organization.

**Figure 3:**
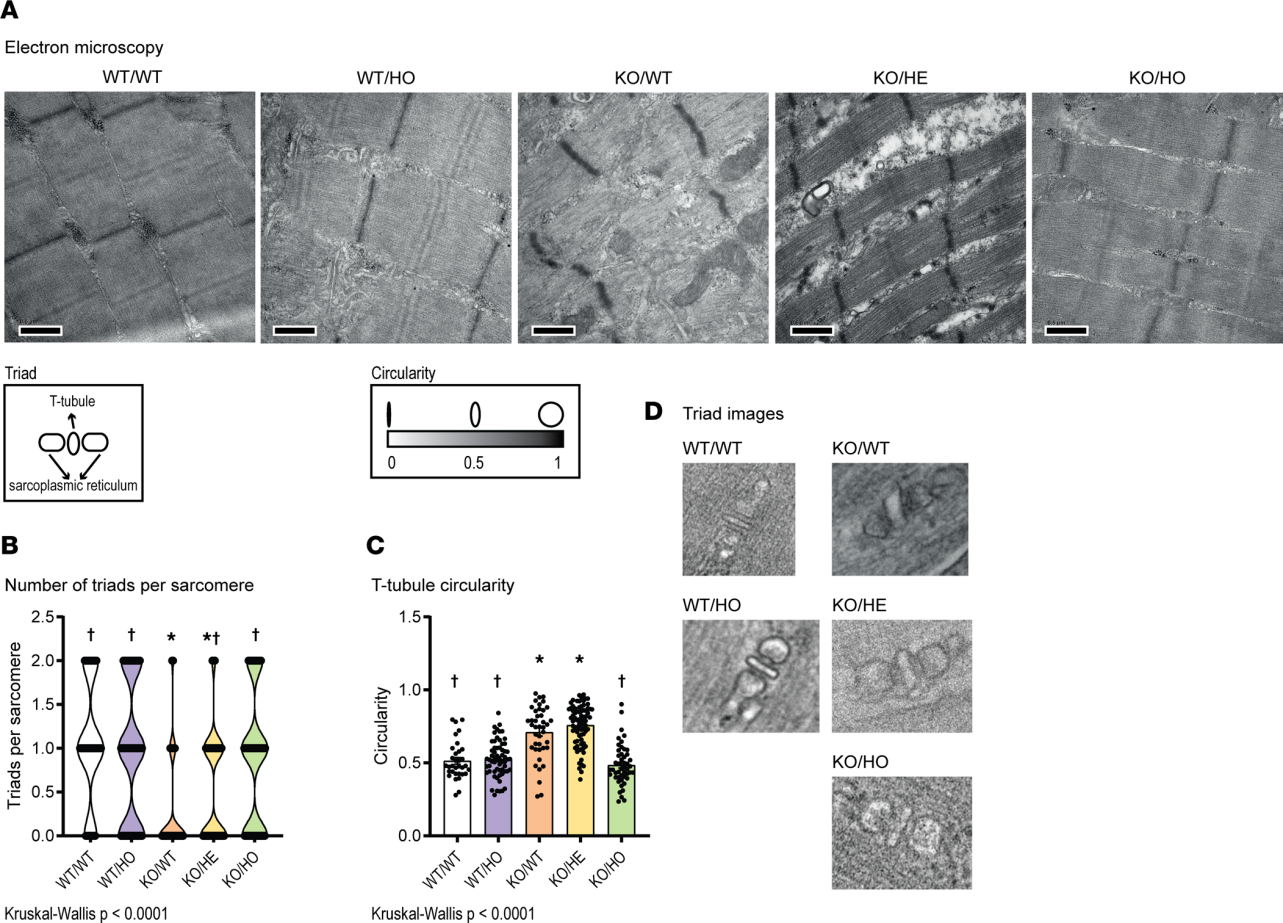
Inhibiting PI3KC2β activity rescues *Mtm1^–/Y^* muscle ultrastructural defects. (**A**) Ultrastructural analysis by electron microscopy of TA muscles from all the analyzed genotypes at 5 weeks of age. Scale bars: 0.5 μm. (**B**) Violin plot showing the number of identifiable triads per sarcomere; each dot represents a sarcomere with 0, 1, or 2 recognizable triads (1 ≤ *n* ≤ 3; *n* sarcomere >39). (**C**) Quantification of T-tubule circularity (1 ≤ *n* ≤ 3; *n* T-tubules >33). (**D**) Representative images of triads from each genotype analyzed. *P < 0.05 vs. WT/WT; ^†^*P* < 0.05 vs. KO/WT by Kruskal-Wallis test (**B** and **C**).

As specific inhibition of PI3KC2β kinase activity was linked to the normalization of muscle atrophy and muscle force and correlated with a strong improvement in muscle histology, electron microscopy was used to assess the intracellular organization of myofibers in greater detail. The internal organization of sarcomeres and their alignment were strongly disrupted in the KO/WT mice at 5 weeks, with Z-line misalignment and disappearance of the A-band and I-band (Figure 3A). The ultrastructural organization of sarcomeres appeared normal in the KO/HO mice. The KO/HE mice displayed an improvement of the sarcomere structure, with some misalignment and enlarged intermyofibrillar space. A main cause of myotubular myopathy was proposed to be triad disruption and dysfunction (23, 31). Triads are composed of 2 junctional sarcoplasmic reticula in contact with a T-tubule and control excitation-contraction coupling. Myofibers from the KO/WT mice showed a strong decrease in the number of recognizable triads per sarcomere and an increased T-tubule circularity (Figure 3, B–D). These parameters were normal in the KO/HO myofibers. KO/HE myofibers showed a partial rescue in the number of triads per sarcomere but no improvement in T-tubule circularity. Overall, the kinase-dead *Pik3c2b* mutation in the homozygous state fully rescues sarcomere and triad organization, while the heterozygous mutation provides a partial rescue effect.

### Rescue of β1 integrin intracellular accumulation with kinase-dead PI3KC2β.

**Figure 4:**
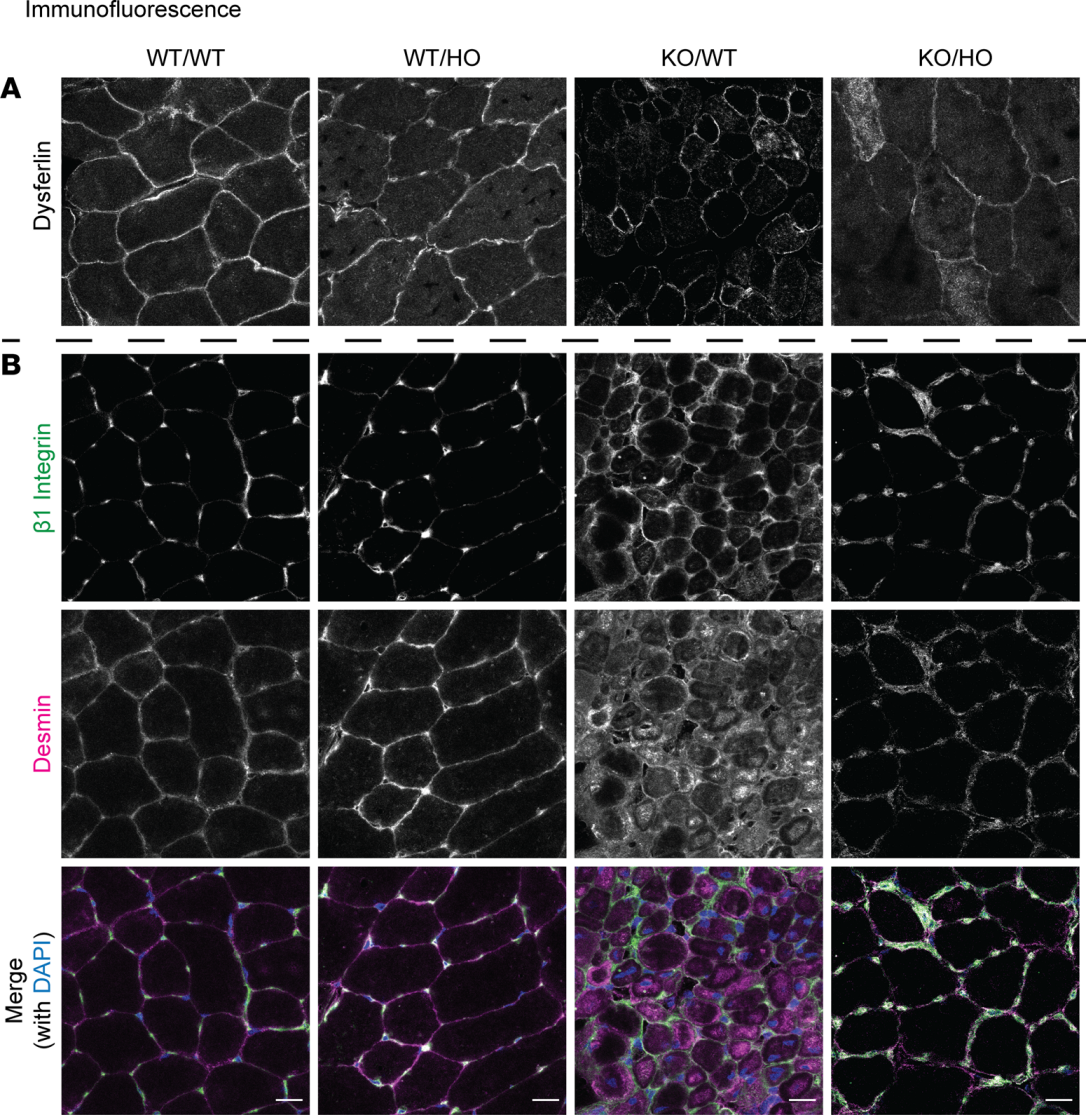
Mislocalization of β1 integrin but not dysferlin is improved in *Mtm1^–/Y^* muscle upon PI3KC2β inactivation. (**A**) Immunolabeling of dysferlin and (**B**) coimmunostaining of β1 integrin and desmin in transverse TA muscle sections at 5 weeks of age. Scale bars: 10 μm.

Myotubular myopathy is linked to defects in membrane trafficking and organelle positioning. Dysferlin localization was assessed as a marker of membrane trafficking and T-tubule biogenesis, and was previously reported to be abnormal in different forms of CNM (22, 32). Dysferlin accumulated inside some myofibers in both KO/WT and KO/HO mice (Figure 4A). Next, desmin was examined, as it binds to myotubularin and is an important cytoskeleton for organelle positioning in muscle (24). KO/WT myofibers displayed strong desmin accumulation, while this was not noted in the KO/HO mice (Figure 4B). In addition, β1 integrin localization was investigated, as it was reported to be defective in KO/WT mice, which could explain the CNM histology with small rounded myofibers and increased intermyofiber space (33). Moreover, the *Drosophila* orthologs of PI3KC2β and MTM1 control β1 integrin recycling in a PI-dependent manner (26). The intracellular accumulation of β1 integrin in the KO/WT mice was rescued in the KO/HO mice (Figure 4B).

### The rescue of the Mtm1-KO muscle phenotypes correlates with normalization of PtdIns3P levels and mTORC1 activation.

**Figure 5:**
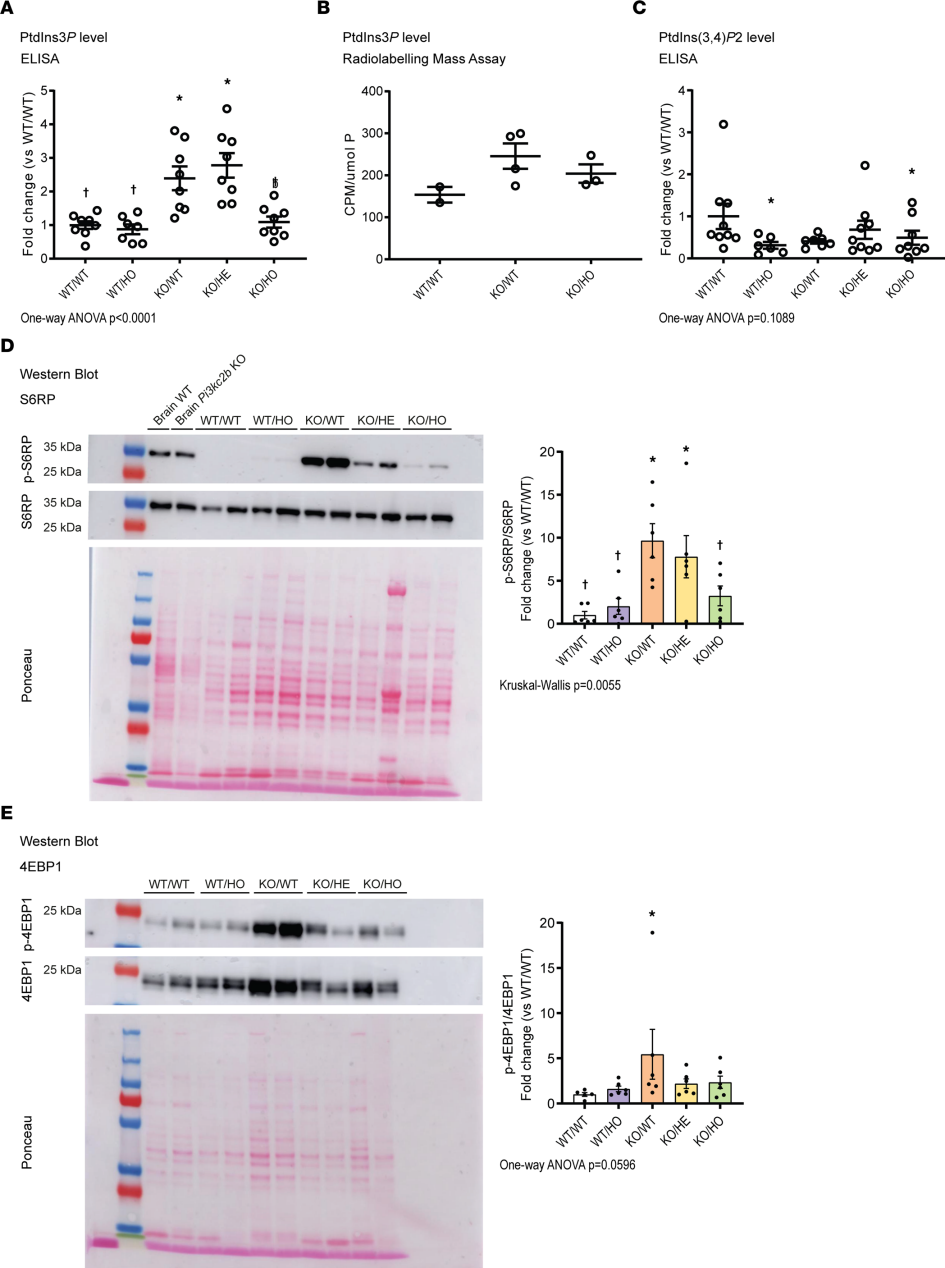
Increased PtdIns3P level and mTORC1 activity in *Mtm1^–/Y^* is restored by inactivating PI3KC2β. Quantification of PtdIns3P levels by (**A**) ELISA (7 ≤ *n* ≤ 8) and (**B**) radiolabeling mass assay (2 ≤ *n* ≤ 4). (**C**) Quantification of PtdIns(3,4)P*_2_* levels by ELISA (6 ≤ *n* ≤ 9). (**D**) Western blotting of TA muscle extracts (and brain when specified) probed with anti–p-S6RP and anti-S6RP antibodies, each normalized to its ponceau staining, and quantified as a ratio (*n* = 6). (**E**) Western blotting of TA muscle extracts probed with anti–p-4EBP1 and anti-4EBP1 antibodies, each normalized to its ponceau staining, and quantified as a ratio (5 ≤ *n* ≤ 6). *P < 0.05 vs. WT/WT; ^†^*P* < 0.05 vs. KO/WT by 1-way ANOVA test (**A**, **C**, and **E**) or Kruskal-Wallis test (**D**).

To asses whether the phenotypic rescue of KO/WT mice with the kinase-dead PI3KC2β is linked to normalization of the PtdIns3P level, we measured PtdIns3P levels in GAS muscles from the different groups of mice with 2 different methods, ELISA and a specific mass assay. As expected, there was a 2.5-fold increase in PtdIns3P in KO/WT muscles compared with WT muscles by ELISA (Figure 5A), and a similar trend was observed with the mass assay (Figure 5B). Both methods indicated that the PtdIns3P level was normalized in the KO/HO mice, strongly suggesting that changes in the level of this lipid is at the basis of the myopathy rescue. As PI3KC2β also phosphorylates PtdIns4P, we looked at the PtdIns(3,4)P_2_ level by ELISA in GAS muscles. As expected, loss of PI3KC2β induced a decrease in the PtdIns(3,4)P_2_ level in WT/HO mice (Figure 5C), and similarly in KO/HO mice. While there was a tendency to decrease in *Mtm1*-KO mice, it was not statistically different from WT. PtdIns(3,4)P_2_ is not a substrate of MTM1 in vitro (9–11).

As PtdIns3P and PI3KC2β are known to regulate autophagy (20, 34), we measured the activity of the mTORC1 pathway by Western blotting. S6RP and 4EBP1, two proteins downstream of mTORC1, were hyperphosphorylated in KO/WT muscles compared with WT (Figure 5, D and E). See complete unedited blots in the supplemental material. mTORC1 overactivity in *Mtm1*-KO mice was associated with the observed muscle weight loss (Figure 1) and fiber hypotrophy (Figure 2). The normalization of mTORC1 activity in the KO/HO mice correlated with a rescue of the muscle mass and fiber size. Thus, the myopathy rescue correlates with a normalization of mTORC1 activity and PtdIns3P level but not of the PtdIns(3,4)P_2_ level. These results suggest that the rescue is achieved through PtdIns3P-mediated mTORC1 pathway regulation.

Among class II PI3Ks generating PtdIns3P and PtdIns(3,4)P_2_, class IIA and IIB expression is detectable in skeletal muscle (Supplemental Figure 1C). The expression of *Pik3c2b* mRNA was significantly increased in the KO/HO mice, and similarly *Pik3c2a* expression was also increased, suggesting a compensatory mechanism for the lack of PI3KC2β kinase activity (Supplemental Figure 1D). Unexpectedly, an increase in expression of MTM1 was also noted in muscles from the WT/HO mice (Supplemental Figure 1E). In addition, DNM2 levels were increased 2-fold in the KO/WT mice (Supplemental Figure 1F), as previously reported (28). DNM2 downregulation was validated as an efficient therapeutic strategy in the KO/WT mice (28, 35). We found that the DNM2 level was not normalized upon PI3KC2β kinase inhibition in the KO/HO mice. Overall, the correction of the KO/WT muscle defects correlates with a normalization of the PtdIns3P level but not of the DNM2 level.

### Specific inhibition of PI3KC2β phospholipid kinase activity does not rescue BIN1-related CNM.

**Figure 6:**
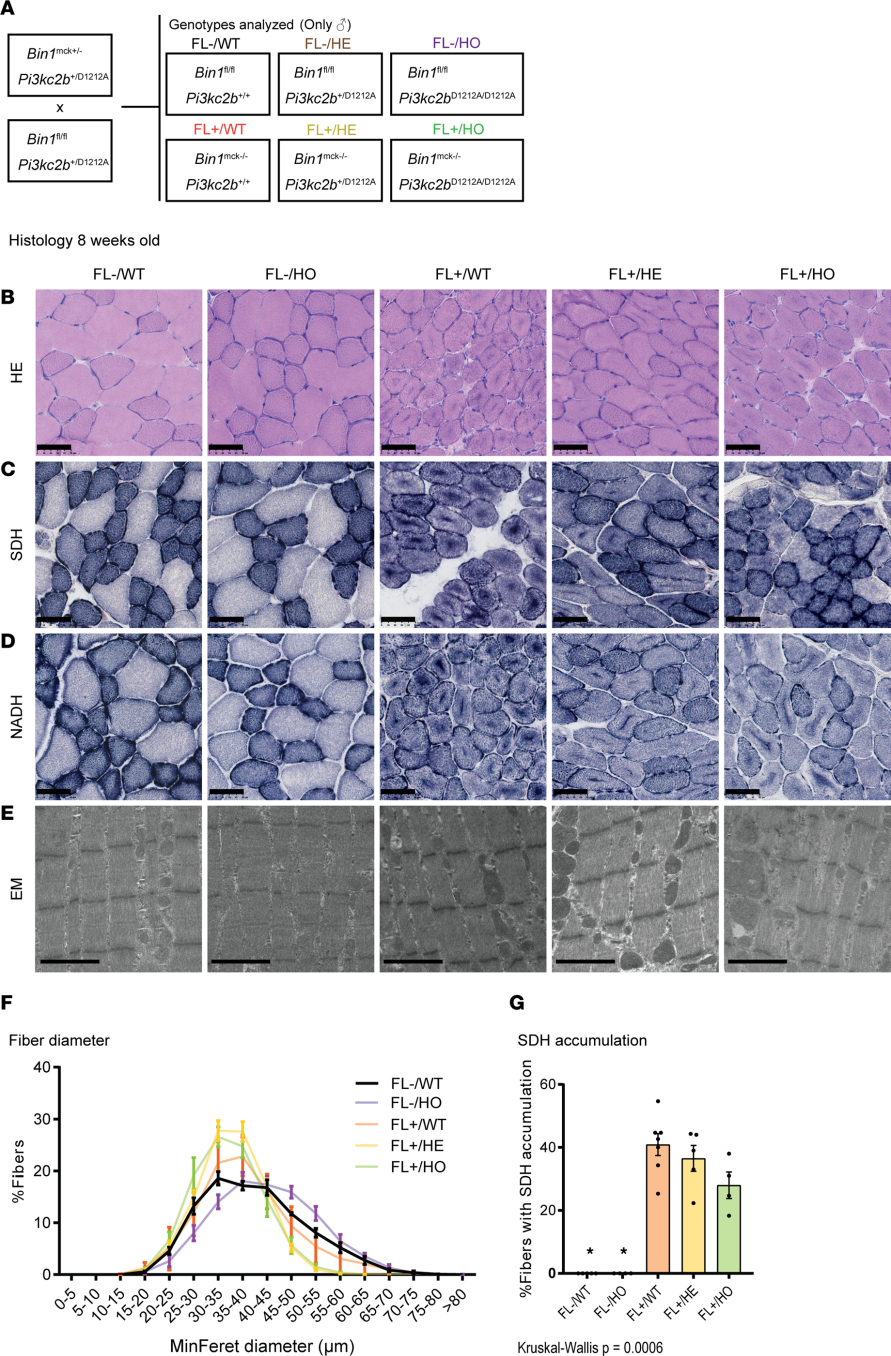
Inactivation of PI3KC2β is not a common therapy for different CNM forms. (**A**) Genotypes analyzed after intercrossing *Bin1*^mck–/–^ and *Pik3c2b*^D1212A^ mice. (**B**–**D**) Histological sections from 8-week-old animals stained with (**B**) H&E and for (**C**) SDH and (**D**) NADH. Scale bars: 50 μm. (**E**) Muscle ultrastructure determined by electron microscopy. Note the mitochondrial defects still present in FL+/HO animals. Scale bars: 2 μm. (**F**) Quantification of fiber diameter (*n* = 4). (**G**) Percentage of fibers with internalized SDH staining (4 ≤ *n* ≤ 7). *P < 0.05 vs. FL+/WT by Kruskal-Wallis test (**G**).

As the PI3KC2β kinase-dead mutation was normalizing all muscle phenotypes from *Mtm1*-KO mice, we tested whether this strategy is effective for another form of CNM linked to mutations in amphiphysin 2 (*BIN1*). We took advantage of the *Bin1*^mck–/–^ mouse that faithfully reproduces the histopathology of BIN1-CNM patients (36). In this model, the floxed *Bin1* exon 20 is deleted upon Cre recombinase expression under the muscle creatine kinase (*MCK*) promoter specific for skeletal and cardiac muscles. We analyzed 5 groups of mice: 2 control groups, including *Bin1*-floxed mice with WT *Pik3c2b* locus (FL–/WT) or with the homozygous *Pik3c2b*^D1212A^ (FL–/HO); the *Bin1*^mck–/–^ mice expressing the Cre recombinase (FL+/WT); and *Bin1*^mck–/–^ mice either heterozygous (FL+/HE) or homozygous (FL+/HO) for *Pik3c2b*^D1212A^ (Figure 6A). While there were no strong motor defects in the FL+/WT mice, they displayed smaller myofibers with central accumulation of SDH and NADH-TR oxidative staining, reflecting mitochondria mispositioning (Figure 6, B–D, F, and G). Heterozygous or homozygous kinase-dead *Pik3c2b* did not rescue the fiber size nor the oxidative accumulation of the FL+/WT mice. Electron microscopy imaging showed enlarged mitochondria in the disease model FL+/WT and the compound heterozygous or homozygous kinase-dead *Pik3c2b* mice (Figure 6E). In conclusion, while the PI3KC2β kinase-dead mutation rescues the *Mtm1*-KO mouse model for myotubular myopathy linked to MTM1 loss, it does not improve the *Bin1*^mck–/–^ mouse model for autosomal recessive CNM.

## Discussion

### Specific inhibition of PI3KC2β kinase activity rescues myotubular myopathy in mice.

Here we show that inactivation of the kinase activity of PI3KC2β is sufficient to efficiently rescue the motor behavior, muscle atrophy, muscle weakness, the histopathology, and sarcomere and triad organization defects of the *Mtm1*-KO mouse model of myotubular myopathy. These findings are in accordance with the rescue of MTM1-related phenotypes upon full loss of class II PI3K or PI3KC2β in *Drosophila* and mice, respectively (22, 26). PI3KC2β may produce PtdIns3P and/or PtdIns(3,4)P_2_ (16, 30). PtdIns3P is the main substrate of myotubularin, while PtdIns(3,4)P_2_ is not a recognized substrate (11) (Figure 5C). We thus propose that the therapeutic effect is mainly based on the modulation of PI levels, most probably through normalization of the PtdIns3P level, as shown in muscle (Figure 5A). These results also reinforce the hypothesis from Sabha and colleagues that PI3KC2β and PI3KC3 kinases act on separate PtdIns3P pools involved in different cellular pathways. Indeed, ablation of PI3KC3 worsens the *Mtm1*-KO phenotypes, while reducing PtdIns3P through inactivation of PI3KC2β rescues these phenotypes. We hypothesize that PtdIns3P is a positive regulator of the mTORC1 pathway, as its normalization was consistent with mTORC1 activity rebalancing.

The *Drosophila* orthologs of MTM1 and PI3KC2β were found to coregulate the trafficking of β1 integrin and MTM1 loss of function causes β1 integrin accumulation in endosomes in cells and muscles (18, 26, 33). β1 Integrin is an important linker between the cell and extracellular matrix and a key regulator of mechanotransduction. β1 Integrin is abnormally accumulated inside myofibers in the *Mtm1*-KO mouse and patients, most probably leading to the small, rounder myofibers and intracellular disorganization typical of myotubular myopathy (26, 33). β1 Integrin localization was rescued with the *Pik3c2b* kinase-dead mutant. Overall, we hypothesize that inactivation of PI3KC2β kinase activity normalizes PtdIns3P homeostasis, mTORC1 activity and β1 integrin trafficking, leading to the amelioration of cellular defects and rescue of the muscle structure and function. This is in line with some recent findings in cells supporting the notion that PI3KC2β controls integrin turnover (37).

Here, we identified the kinase activity of PI3KC2β as a main therapeutic target for myotubular myopathy. A limitation of our study was the use of a germline inhibition of PI3KC2β, while patients would be treated postsymptomatically. Until now, no specific kinase inhibitor for PI3KC2β has been developed and published to our knowledge. This work promotes the development of specific inhibitors to further treat this devastating disease. Of note, our data underline a dose-response effect, as the kinase-dead mutation in the heterozygous state only led to a partial rescue and extended lifespan in approximately half of the *Mtm1*-KO mice. This suggests that a drug-based treatment reaching 50% inhibition could already provide some improvement and that a stronger inhibition of PI3KC2β kinase activity will be required to reach a higher clinical significance.

### Targeting PI3KC2β is not a common therapy for several forms of CNM.

Specific inhibition of PI3KC2β kinase function does not ameliorate the CNM-like histopathological features displayed by the *Bin1*-KO mice, a faithful model for the autosomal recessive CNM form. Thus, targeting PI3KC2β does not appear to represent a common therapy for several CNM forms. While BIN1 was found to bind to MTM1, it does not regulate its PI phosphatase activity, supporting the idea that BIN1-CNM is not due to a defect in PI homeostasis (38). Notably, overexpression of human BIN1 rescued the β1 integrin defects and the overall phenotype of the *Mtm1*-KO mouse, supporting the idea that normalization of β1 integrin trafficking is a common effect of both therapies (33). PI3KC2β and BIN1 would thus regulate β1 integrin trafficking through different mechanisms.

MTM1 is part of a large family of proteins mutated in different neuromuscular diseases. Deletion of *Pik3c2b* in the *Mtmr13*-KO mouse model of Charcot-Marie-Tooth demyelinating neuropathy (CMT4B2) was unable to ameliorate the myelin outfoldings around axons of the sciatic nerves (39). Taken together, these findings suggest that the targeting of PI3KC2β represents a therapy specifically for MTM1 myopathy.

Downregulation of DNM2 has been shown to efficiently rescue the phenotypes of mouse models for MTM1, BIN1- and DNM2-related CNM, representing a potential common therapy for several CNM forms (35, 40, 41). Upon PI3KC2β inhibition, we found no normalization of DNM2 level that is increased in *Mtm1*-KO mouse and patient muscles. We conclude that either the therapeutic effect of the modulation of PI3KC2β and DNM2 is mediated through different pathways, suggesting applying both therapeutic strategies may have a synergistic effect, or that PI3KC2β kinase inhibition affects endosomal pathways downstream of DNM2.

## Methods

### Animal models and experimental design.

The mouse lines used in this study have been described previously: *Mtm1^–/Y^* 129 Sv/Pas (8), *Pik3c2b*^D1212A^ C57BL/6N (30), and *Bin1*^mck–/–^ C57BL/6J (36). Male mice were analyzed, as the *Mtm1* gene is X-linked and only the *Mtm1^–/Y^* but not the *Mtm1^+/–^* females are affected. Mouse crosses and groups studied are detailed in Figures 1A and 6A. Mice were kept on a 12-hour light/dark cycle with free access to standard food and water. Lifespan and body weight were monitored during this study from 4 to 12 weeks, hanging tests performed at 4 weeks, and muscle force and weight, histology, and electron microscopy at 5 weeks old for the MTM1 study, while the muscle investigations were done at 8 weeks for the BIN1 study.

### Muscle contractile properties.

TA muscle force was assessed by measuring in situ muscle contraction after sciatic nerve stimulation using the Complete1300A mouse Test System (Aurora Scientific) as described previously (41). Briefly, mice were anesthetized by sequential intraperitoneal injections of Domitor/fentanyl mix (2/0.28 mg/kg), diazepam (8 mg/kg), and fentanyl (0.28 mg/kg). The TA tendon was then detached and tied to an isometric transducer. The sciatic nerve was stimulated with pulses of 1–125 Hz and the absolute maximal force was determined. The specific maximal force was calculated by dividing absolute maximal force by the TA muscle weight.

### Muscle histology.

TA muscles were dissected and frozen in liquid nitrogen–cooled isopentane and stored at –80°C. Transverse cryosections (8 μm) were then stained for SDH and NADH and with H&E. Tissue sections were observed using a Hamamatsu 322 NanoZoomer 2HT slide scanner.

### Ultrastructural analysis.

Muscle ultrastructure was determined by transmission electron microscopy. Briefly, TA muscles were fixed in 2.5% paraformaldehyde (Electron Microscopy Sciences), 2.5% glutaraldehyde (Electron Microscopy Sciences), and 50 mM CaCl_2_ (Sigma-Aldrich) in cacodylate buffer (0.1 M, pH 7.4; Sigma-Aldrich). Muscles were then postfixed in 1% osmium tetroxide in 0.1 M cacodylate buffer for 1 hour at 4°C. Samples were then incubated with 5% uranyl acetate for 2 hours at 4°C. Muscles were embedded in Epon 812 and ultrathin sections were cut at 70 nm and contrasted with uranyl acetate and lead citrate. A Philips CM12 electron microscope with a Gatan OneView camera was used to observe.

### PtdIns3P mass assay.

Total PtdIns3P levels were quantified by a specific mass assay as previously described (42). Briefly, 30 mg of frozen muscle was homogenized using a FastPrep homogenizer (MP Biomedical) in 1 mL of methanol. Total lipid extraction was performed according to the Bligh and Dyer method. PtdInsP was then purified by thin layer chromatography and submitted to lipid kinase assays in the presence of recombinant PIKfyve and [γ-^32^P]ATP (PerkinElmer) to specifically transform PtdIns3P into PtdIns(3,5)P_2_. The radioactivity incorporated into PtdIns(3,5)P_2_ (proportional to the PtdIns3P present in the sample) was quantified using a Typhoon imaging system (GE Healthcare).

### PtdIns3P and PtdIns(3,4)P_2_ ELISAs.

GAS muscles were dissected and kept at –80°C. To extract the lipids, muscles were ground into fine powder under liquid nitrogen using a mortar and mixed with 3 mL of 5% trichloroacetic acid with 1 mM EDTA. After centrifugation, the supernatant was discarded and 3 mL of MeOH/CHCl_3_ (2:1) was added to extract neutral lipids. After centrifugation, the pellet was then incubated with 2.25 mL of MeOH/CHCl_3_/12N HCl (80:40:1) to extract acidic lipids. The supernatant was then mixed with 0.75 mL of CHCl_3_ and 1.35 mL of 0.1N HCl to collect the organic phase. The organic phase containing the lipids was dried in a vacuum dryer for 1 hour at room temperature. PtdIns3P level was determined using a PI(3)P Mass ELISA (Echelon Biosciences). PtdIns(3,4)P_2_ level was determined using a PI(3,4)P_2_ Mass ELISA (Echelon Biosciences). The day of the ELISA experiment, lipids were rehydrated in PBS-T buffer available from the commercial kit. ELISA was performed following the manufacturer’s instructions.

### Immunofluorescence.

TA transversal sections were immunostained as described previously (41). The following primary antibodies were used at the indicated dilutions: anti-desmin (sc-23879, Santa Cruz Biotechnology; 1:100), anti–β1 integrin (MAB1997, Chemicon; 1:200), and anti-dysferlin (ab124684, Abcam; 1:150). The secondary antibodies used were goat anti-rabbit–Alexa Fluor 488 (A11008, Thermo Fisher Scientific), goat anti-rabbit–Alexa Fluor 647 (A21237, Thermo Fisher Scientific), and goat anti-rat–Alexa Fluor 488 (A11006, Thermo Fisher Scientific) diluted 1:200. Images were acquired in a Leica SP8X microscope.

### Western blotting.

TA muscles were lysed in RIPA buffer supplemented with 1 mM PMSF, 1 mM sodium orthovanadate, 5 mM sodium fluoride, and 1× protease inhibitor cocktail using a Precellys CK14 Lysing Kit (Bertin Technologies). Protein concentrations were calculated using the DC Protein Assay Kit (Bio-Rad). Muscle extracts (10 μg per lane) were separated in 10% polyacrylamide gels, made using a TGX FastCast Acrylamide Kit (Bio-Rad). The proteins were transferred onto nitrocellulose membranes using a Trans-Blot Turbo Transfer System (Bio-Rad). Membranes were blocked for 45 minutes in TBS containing 5% nonfat dry milk and 0.1% Tween 20. The following primary antibodies were diluted 1:1000 in TBS containing 5% BSA and 0.1% Tween 20 and incubated overnight at 4°C: rabbit anti–p-S6RP (2211, Cell Signaling Technology), rabbit anti-S6RP (2217, Cell Signaling Technology), rabbit anti–p-4EBP1 (9459, Cell Signaling Technology), and rabbit anti-4EBP1 (9644, Cell Signaling Technology). Subsequently, membranes were incubated with horseradish peroxidase–coupled goat anti-rabbit secondary antibody (112-036-045, Jackson ImmunoResearch). Proteins were revealed with the SuperSignal West Pico PLUS Chemiluminescent Substrate (Thermo Fisher Scientific), and visualized using an Amersham Imager 600 (GE Healthcare Life Sciences). Ponceau S staining served as loading control.

### Statistics.

Groups of mice compared and genetically crossed to obtain them are defined in Figures 1A and 6A. All experiments were performed and analyzed in a blinded manner and the investigators were unaware of the genotype of the mice. No samples were excluded from the statistical analysis. The normal distribution of the data was assessed using the Shapiro-Wilk test. For normally distributed data, the significance of changes was examined by 1-way ANOVA for comparison of more than 2 groups. In case of not normally distributed data, the Kruskal-Wallis test was performed to analyze more than 2 groups. A *P* value of less than 0.05 was considered significant. Data are presented as mean ± SEM. Number of replicates is indicated in the dot plots in the figures and corresponds to biological replicates unless stated otherwise in legends.

### Study approval.

Animal care and experimentation were in accordance with French and European guidelines and with the approval of the Com’eth Ethics committee (CEEA-017) of ICS animal facility (approval C67-218-40) (project number APAFIS #22712-2019101416421345).

## Author contributions

JL conceived and supervised the project. XMM, VN, DR, and CK performed mouse experiments. XMM, MG, and DR performed histological and molecular analyses. XMM performed immunofluorescent staining and imaging. XMM, DR, MG, GC, and BP performed and analyzed lipid measurements. BV provided the *Pik3c2b* kinase-dead mouse and other reagents and helped edit the manuscript. XMM, MG, and JL wrote the manuscript.

## Supplementary Material

Supplemental data

## Version 1 03/21/2023

In-Press Preview

## Version 2 05/08/2023

Electronic publication
