# Supplementary material for: Inactivating the lipid kinase activity of PI3KC2β is sufficient to rescue myotubular myopathy in mice
Source: JCI Insight. 2023 May 8;8(9):e151933. doi: 10.1172/jci.insight.151933 (PMC10243799; doi:10.1172/jci.insight.151933)

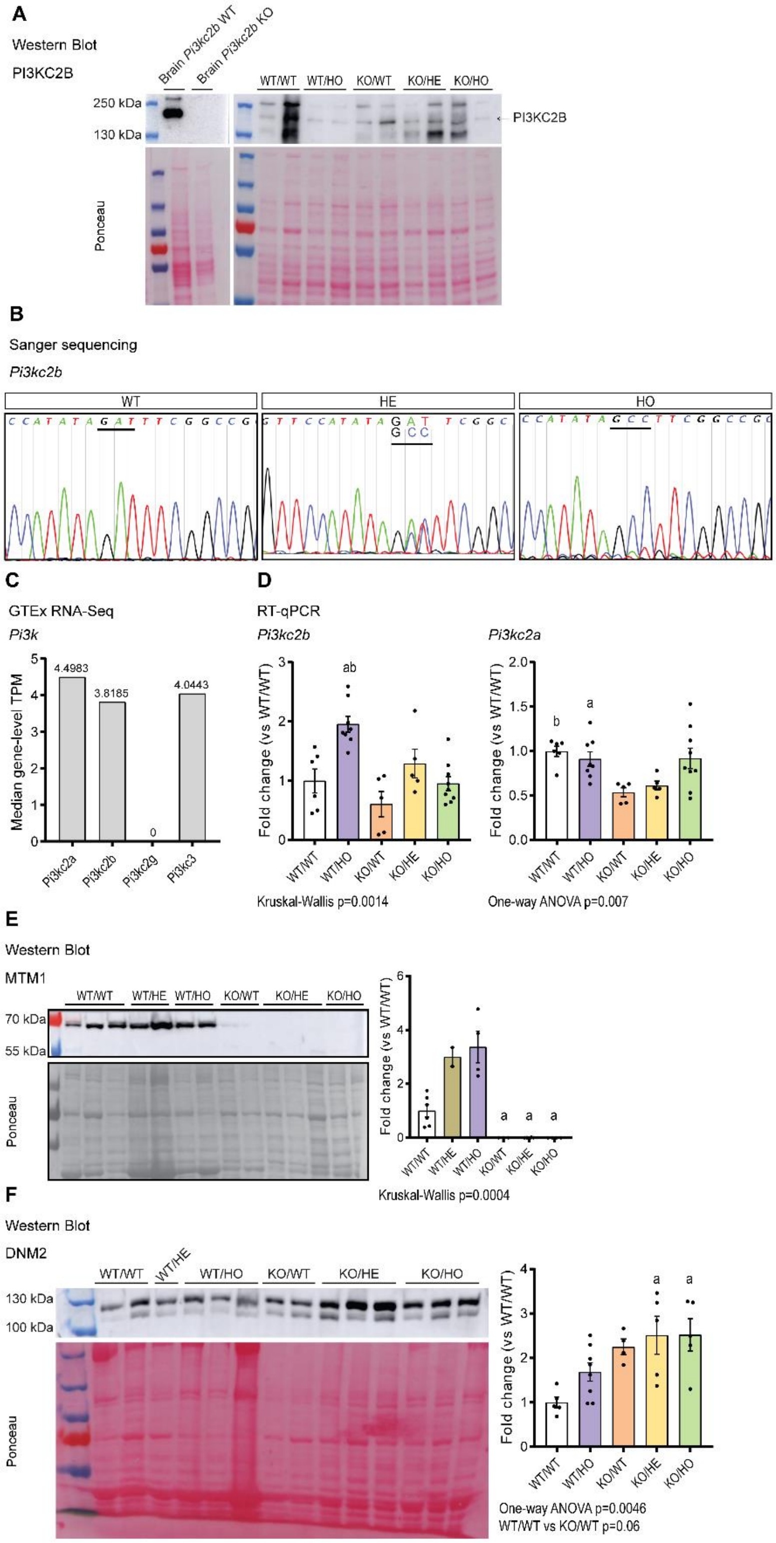

Supplementary Figure 1. Additional data on the characterization of *Pik3c2b* kinase-dead mice

(A) Western blotting of brain and TA muscle extracts probed with anti-PI3KC2β antibody (n=2). **(B)** Chromatopherograms of wild-type, heterozygous or homozygous *Pik3c2b* kinase-dead mice. **(C)** GTEx RNA-seq data showing *Pi3k* class II and III expression in skeletal muscle (n=803). **(D)** RT-qPCR data assessing mRNA levels of *Pik3c2b* and *Pik3c2a* (5≤n≤9). **(E)** Western blotting of TA muscle extracts probed with anti-MTM1 antibody, and quantification as a ratio to Ponceau red (2≤n≤6). **(F)** Western blotting of TA muscle extracts probed with anti-DNM2 antibody, and quantification as a ratio to Ponceau red (4≤n≤8). In the graphs, a: p<0.05 vs WT/WT; b: p<0.05 vs KO/WT. **(D *Pi3kc2b*, E)**: Kruskal-Wallis test. **(D *Pi3kc2a*, F)**: One-way ANOVA test.

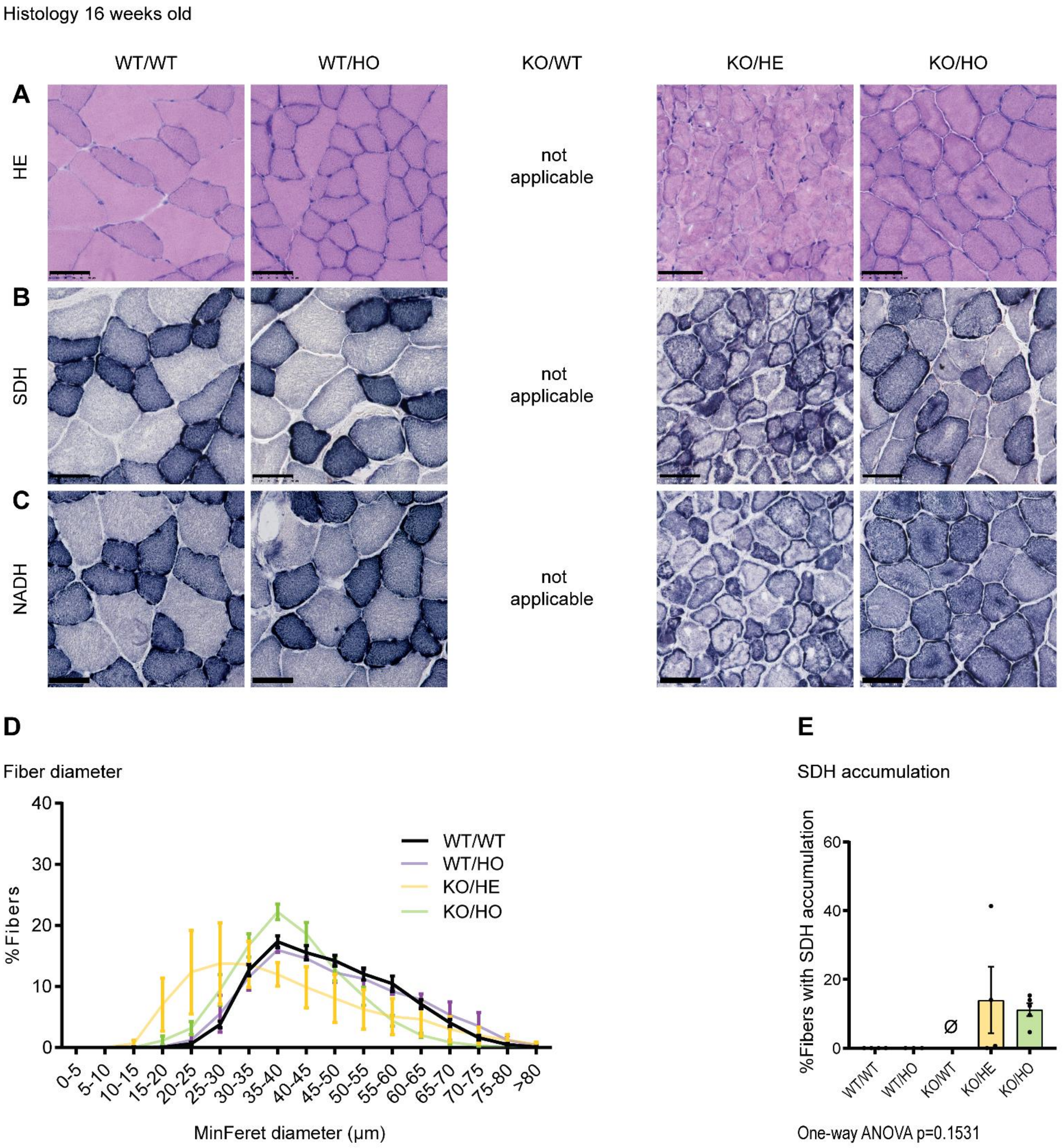

Supplementary Figure 2. Histology at 16 weeks

Muscle sections stained for **(A)** HE, **(B)** SDH and **(C)** NADH. Scale bar = 50 μm. **(D)** Quantifications of fiber diameter. **(E)** Percentage of fibers with SDH staining central accumulation. Note that all KO/WT mice died by 12 weeks of age, before these analyses, and thus histology imaging and quantification is not applicable. All mice (3≤n≤5) analyzed at 16 weeks of age. One-way ANOVA test.

Figure 3

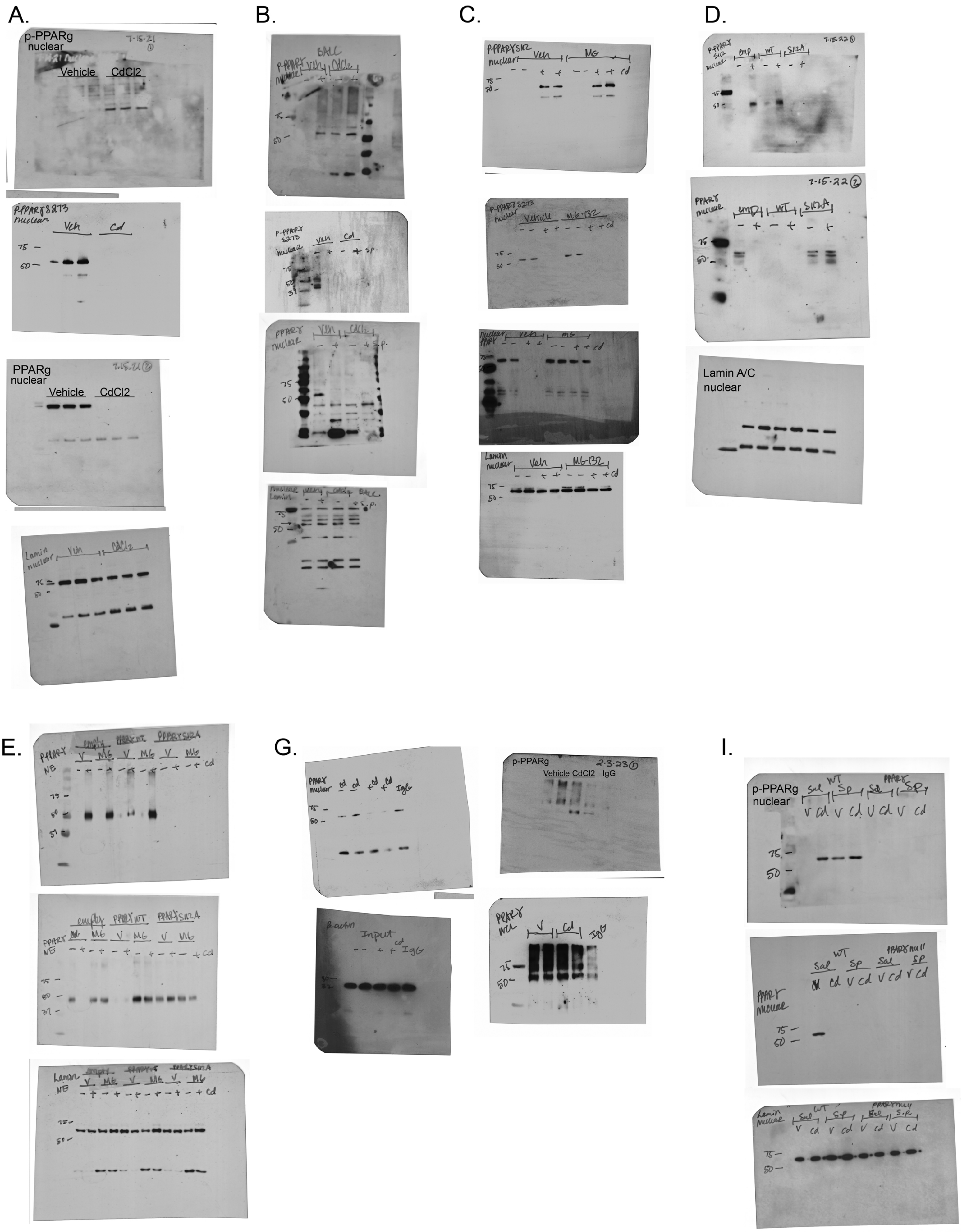
A.
B.
C.
D.
E.
G.
I.
p-PPARg
nuclear
Vehicle
CdCl2
PPARg
nuclear
Vehicle
CdCl2
Lamin A/C
nuclear
p-PPARg
Vehicle
CdCl2
IgG
p-PPARg
nuclear

Figure 4

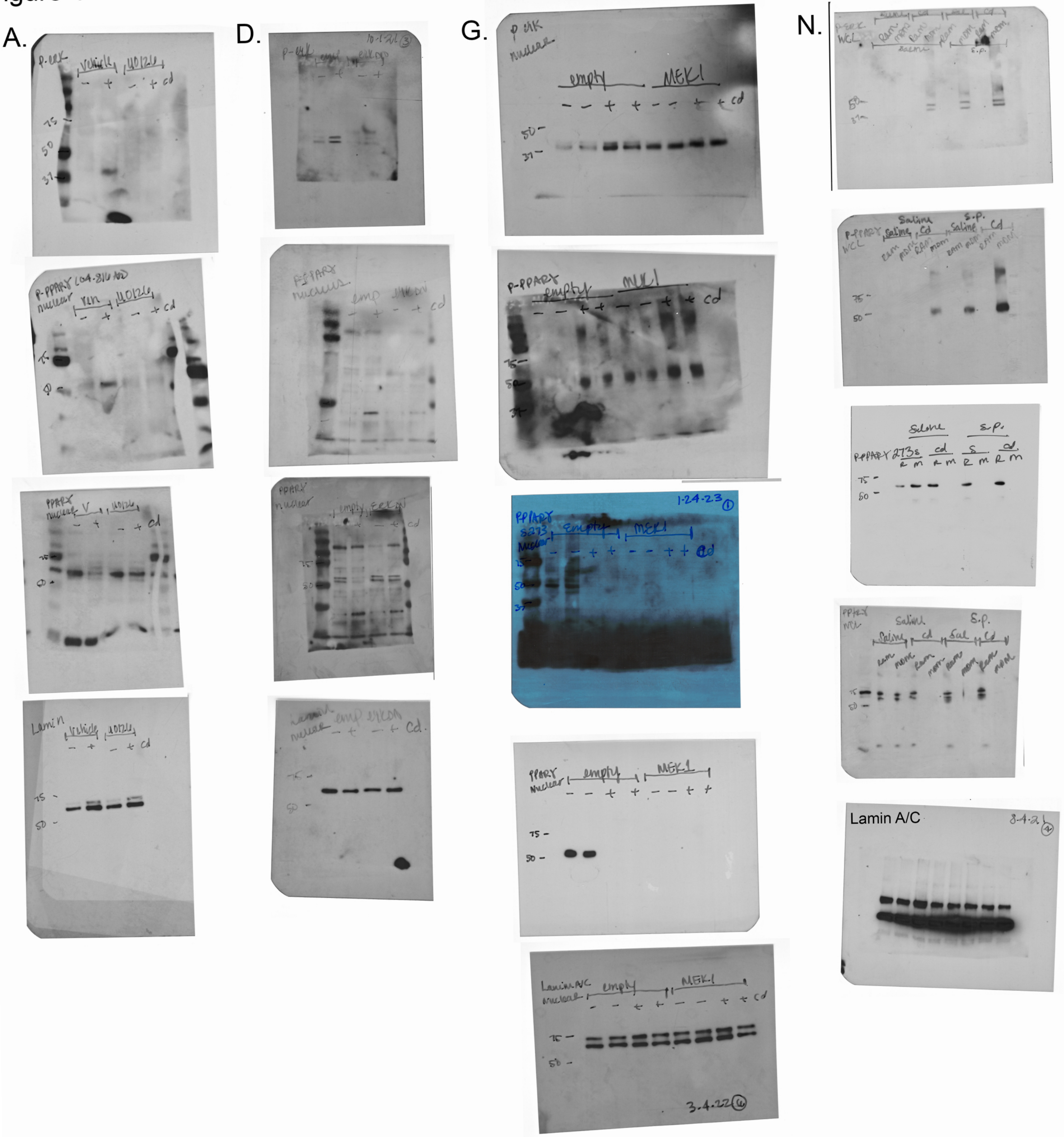

Figure 6

C.

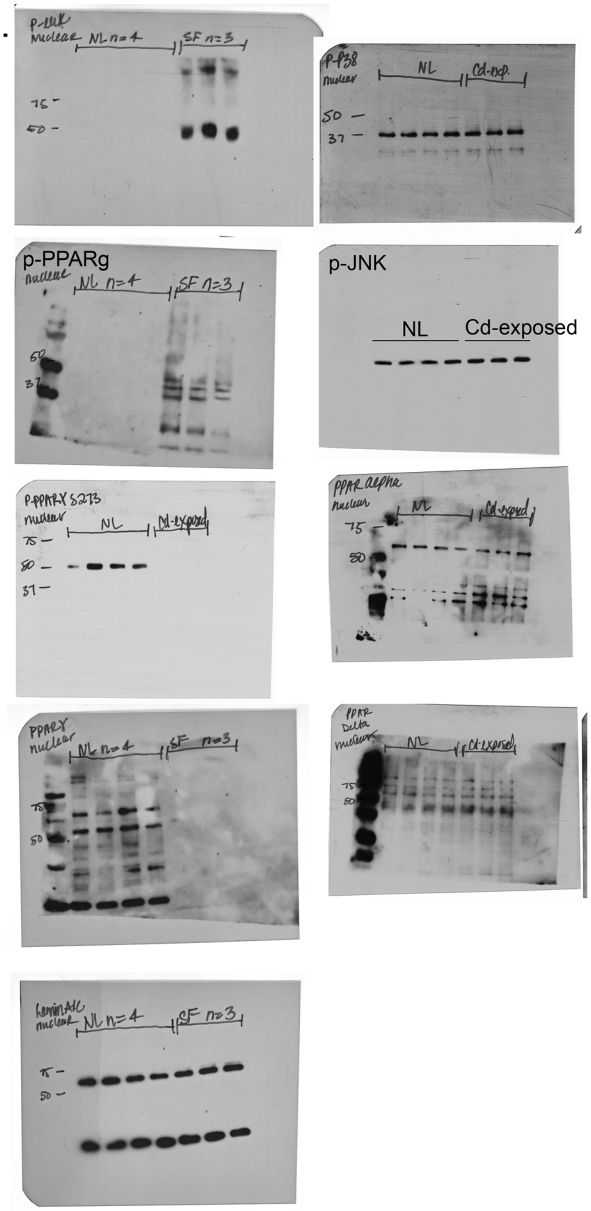

Figure 7

A.

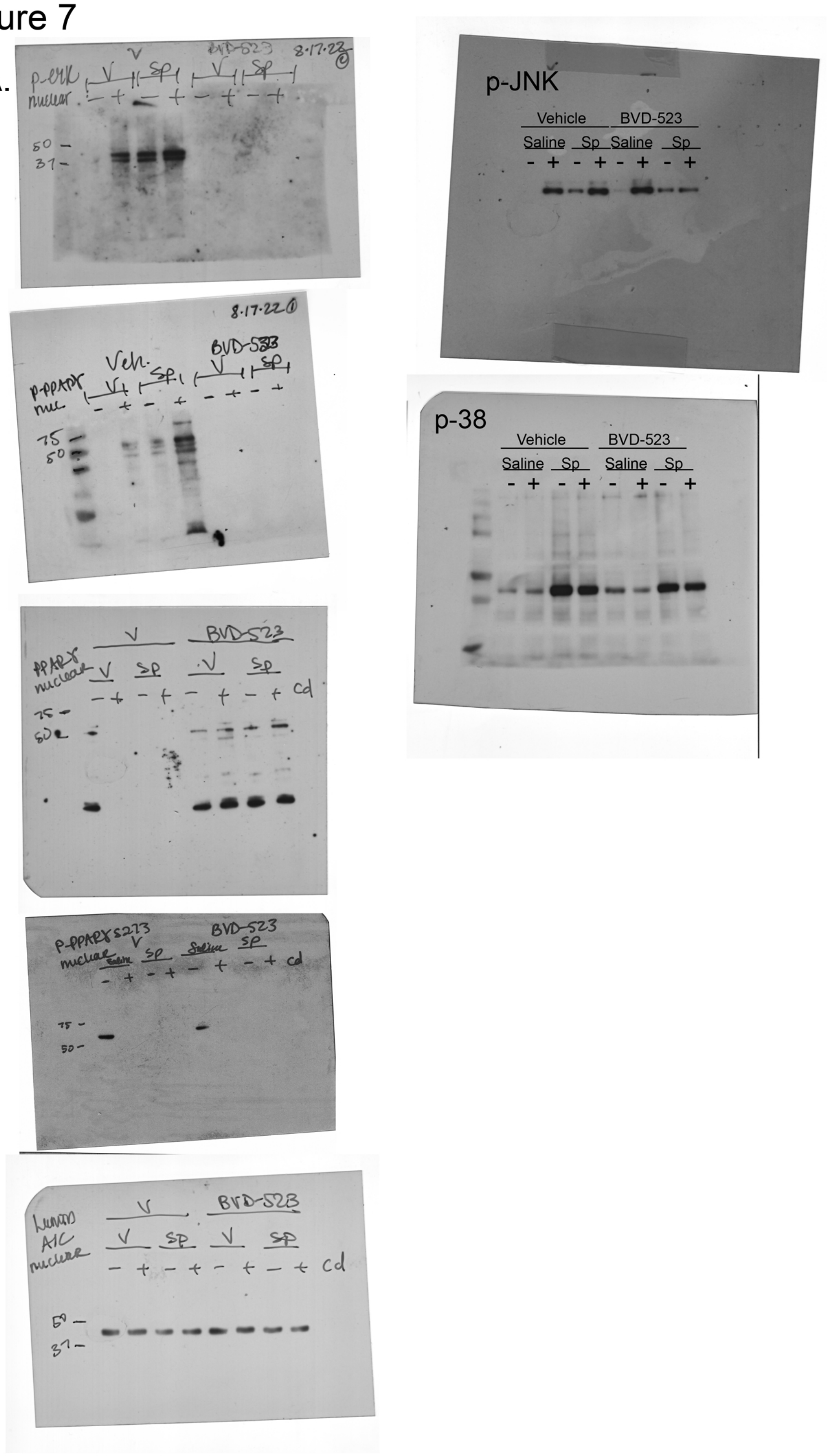

Supplemental Figure 3

N.

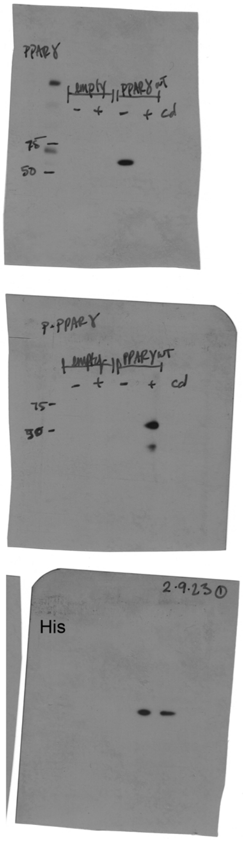

Supplemental Figure 4

E.

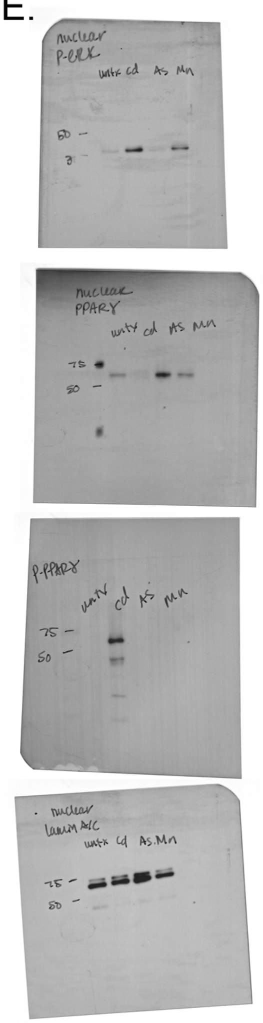

Supplement: Supplemental data [file jciinsight-8-151933-s017.pdf]
